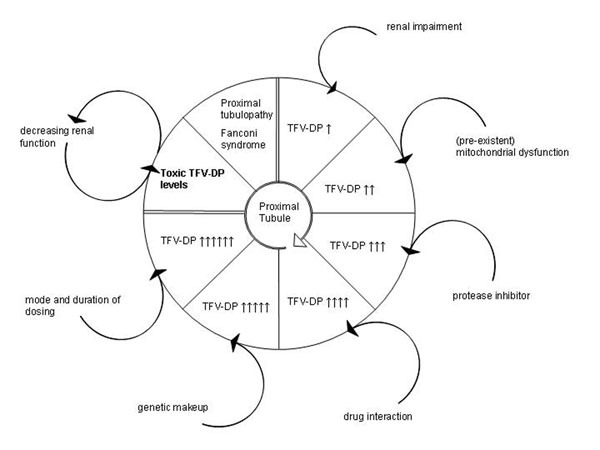# Intracellular tenofovir-diphosphate accumulation in an HIV-infected patient with Fanconi syndrome and osteomalacia

**DOI:** 10.1186/1758-2652-13-S4-P87

**Published:** 2010-11-08

**Authors:** ME Haverkort, BW van der Spek, PT Lips, WAT Slieker, W Bronsveld

**Affiliations:** 1 Academic Medical Center, Meibergdreef 9, Amsterdam, Netherlands; 2 VU University Medical Center, Amsterdam, Netherlands; 3 Medical Center Alkmaar, Alkmaar, Netherlands

## 

We present a patient with tenofovir disoproxil fumarate (TDF)-induced Fanconi syndrome, osteomalacia and concurrent nucleos(t)ide reverse transcriptase inhibitor related CD4 cell decline. Sequentially measured intracellular (ic) tenofovir-diphosphate (TFV-DP) levels were extremely high, with plasma TFV just slightly elevated.

In June 2008 a 53-year-old Caucasian male complained of severe pain in his lower extremities. He was diagnosed with HIV in 1995 and developed some polyneuropathy and severe lipoatrophy after initiation of ART in 1996. In September 2003 TDF 300 mg qd, didanosine (ddI) 250 mg qd and lopinavir-ritonavir (LPV-r) 400/100 mg bid were started. CD4 cells had increased from 136 to 489/mm^3^ by December 2007, but then slowly decreased to 181 despite a persistently undetectable HIV-1 RNA. There was 13 kg weight loss when he developed diabetes mellitus in April 2008. In May 2008 blood and urinalysis were compatible with Fanconi syndrome. Creatinine clearance (CCl) had decreased from 76 (2003) to 44 mL/min (GC). Serum 1,25(OH)-vitamin D was low (20 pmol/L), but 25(OH)-vitamin D and PTH were normal. MRI of feet and knees showed patchy bone marrow oedema without fractures, DXA demonstrated osteopenia and bone biopsy confirmed the diagnosis osteomalacia. While CCl decreased, plasma TFV increased only slightly (from 0.23 in July 2004 to 0.36 mg/mL in September 2008), but ic TFV-DP levels were found to be extremely high, 3630 fmol/10^6^ cells (mean TFV-DP in patients on LPV-r 233.1 fmol/10^6^ cells [1]). Eight weeks after TDF dose reduction and 2 weeks after TDF cessation ic TFV-DP was still high (310 fmol/10^6^ cells), but plasma TFV was undetectable, illustrating the long ic half-life. Ic dideoxy adenosine-triphosphate (ddATP) levels were also high, 123 fmol/10^6^ cells (n 5.06 fmol/10^6^ cells) as were ddI plasma levels (max. 0.304 mg/L). TDF and ddI were replaced by raltegravir/nevirapine and phosphate, calcium and 1,25(OH)2-VitD temporarily supplemented. Two months later symptoms disappeared, CCl improved to 62 mL/min, CD4s increased (286 cells/mm^3^) and oral antidiabetics could be stopped.

This case illustrates the severe clinical impact of protracted unrecognised TDF-related toxicity and is suggestive of a causal relationship between TDF use, concomitant factors and ic TFV-DP accumulation (fig 1). A significant CD4 cell decline in patients with undetectable HIV-RNA on TDF- and/or ddI-containing ART should alert the physician to investigate for NRTI toxicity.

**Figure 1:**